# Azithromycin-Induced Liver Injury in an Asthma Exacerbation Patient With Autoimmune Features

**DOI:** 10.7759/cureus.25447

**Published:** 2022-05-29

**Authors:** Richard Liang, Adesh Ramdass

**Affiliations:** 1 Medicine, New York Institute of Technology College of Osteopathic Medicine, Glen Head, USA; 2 Medicine, Icahn School of Medicine at Mount Sinai, Queens Hospital Center, New York, USA

**Keywords:** drug-induced liver injury, acute hepatitis, asthma exacerbation, autoimmune hepatitis, azithromycin

## Abstract

Drug-induced liver injury (DILI) is one of the leading causes of acute liver failure in the United States. Antimicrobials are the most common class of drugs implicated in this pathology. Although azithromycin has been documented as a relatively safe drug, one of its rare and potentially fatal side effects is DILI. Diagnosing DILI is difficult because it is a diagnosis of exclusion. Autoimmune hepatitis (AIH) may present similarly to DILI, and a liver biopsy may be needed to differentiate between the two conditions. We present a case of azithromycin-induced liver injury in an asthma exacerbation patient with features of AIH.

## Introduction

Azithromycin is a commonly used macrolide antibiotic for both gram-positive and gram-negative bacterial infections [1]. In the treatment of asthma, its anti-inflammatory properties both improve symptoms and reduce the frequency of exacerbations [2]. Some of the common minor side effects of azithromycin include gastrointestinal upset, headache, and dizziness [1]. Significant adverse reactions to azithromycin include QT interval prolongation leading to torsades de pointes, hypersensitivity reactions, and ototoxicity [3-5]. Cases of azithromycin-induced liver injury have also been reported in the literature [6-9]. Despite these uncommon side effects, azithromycin is still listed by the World Health Organization as one of the safest and most effective medications in the current health system [1].

Drug-induced liver injury (DILI) is a rare clinical event but has been noted as one of the leading causes of acute liver failure (ALF) in the United States [10]. DILI usually occurs within six months of starting a new medication [11]. It is a diagnosis of exclusion, relying on obtaining a detailed history along with extensive blood work, hepatobiliary imaging, and a liver biopsy [12]. It is also important to identify the type of DILI that the patient has, whether it is hepatocellular, cholestatic, or mixed, to narrow down differential diagnoses [11]. Hepatocellular DILI has a ratio of the serum alanine aminotransferase (ALT) and alkaline phosphatase (ALP) equal to or above 5, cholestatic DILI has a ratio under 2, and mixed DILI has a ratio between 2 and 5 [11]. Acetaminophen has been noted as the most common hepatotoxic drug to cause ALF, compromising 46% of subjects enrolled in the Acute Liver Failure Study Group (ALFSG) [13]. In terms of overall drug classes implicated in DILI, antimicrobials were the most common culprits, with amoxicillin-clavulanate, isoniazid, and nitrofurantoin being the most common individual agents [10]. Treatment of DILI includes the withdrawal of the offending medication, with 90% of patients recovering [11]. In certain instances, where there are no signs of recovery, liver transplantation may be necessary [13]. Normalization of liver function tests (LFTs) may vary from days to months [11].

Autoimmune hepatitis (AIH) is a chronic inflammatory liver disease caused by a T-cell-mediated immune response targeting the liver [14]. Diagnosis of AIH includes laboratory findings showing elevated LFTs, increased immunoglobulin (Ig)G concentration, and the presence of one or more of the characteristic autoantibodies: antinuclear antibodies (ANA), anti-smooth muscle antibodies (ASMA), and antibodies to liver-kidney microsome type 1 (anti-LKM1) [15]. In addition, histological examination is essential in making a diagnosis of AIH, which would present with interface hepatitis accompanied by plasma cell infiltration and lobular hepatitis [15]. The treatment of AIH is steroid induction therapy followed by maintenance therapy with azathioprine [16]. Clinical response to corticosteroids is also one of the diagnostic characteristics of AIH [14]. Second-line treatment in patients who do not respond to standard treatment involves immunosuppressants such as mycophenolate mofetil [15]. Early identification and management of AIH improve patient outcomes, but like DILI, the diagnosis of AIH requires the exclusion of other etiologies of hepatitis [15,16]. We report a case of azithromycin-induced liver injury in an asthma exacerbation patient who also had some features of AIH.

## Case presentation

A 73-year-old female patient with a significant medical history of hyperlipidemia, hypertension, well-controlled non-insulin-dependent type 2 diabetes mellitus, and asthma presented to the emergency department with a worsening cough and shortness of breath for the past two months. The patient had returned from a vacation in Trinidad four months prior to her presentation. One month before admission, the patient went to an urgent care for the same symptoms, was diagnosed with an asthma exacerbation, and was discharged with prednisone 10 mg oral tablet once daily for five days and azithromycin 500 mg oral tablet once daily for one day, followed by 250 mg oral tablet once daily for the following four days. The patient endorsed that her cough and shortness of breath improved while taking the medications, but after she finished the treatment, the symptoms never fully resolved and progressively worsened over the last two weeks. The patient endorsed that her cough became more productive and that she spiked a fever of 101°F once during this time which had since resolved. The patient also noticed that for the past week, she had dark urine and her skin was itchy. Upon visiting her primary care provider, she was recommended to go to the emergency department to evaluate for possible pneumonia. The patient denied illicit drug use, alcohol use, smoking history, herbal supplement ingestion, and any family or personal history of liver disease. The patient also denied any significant family history of medical conditions. The patient’s home medications included albuterol 108 µg/ACT inhaler two puffs every four hours as needed, amlodipine-olmesartan 5-40 mg oral tablet once daily, atorvastatin 20 mg oral tablet once daily, budesonide-formoterol 160-4.5 µg/ACT inhaler two puffs twice daily, gabapentin 300 mg oral tablet once daily, and glimepiride 4 mg oral tablet twice daily.

**Figure 1:**
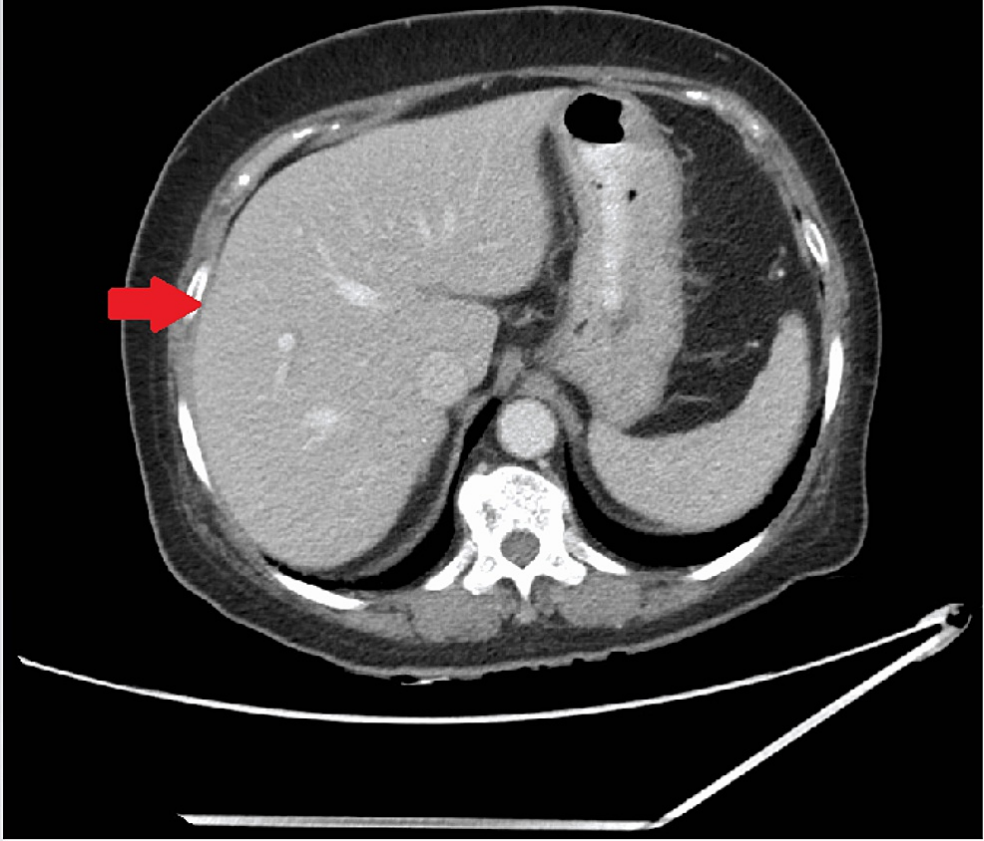
Unremarkable liver visualized on computed tomography of the abdomen and pelvis with no hepatic nodules/lesions noted.

**Figure 2:**
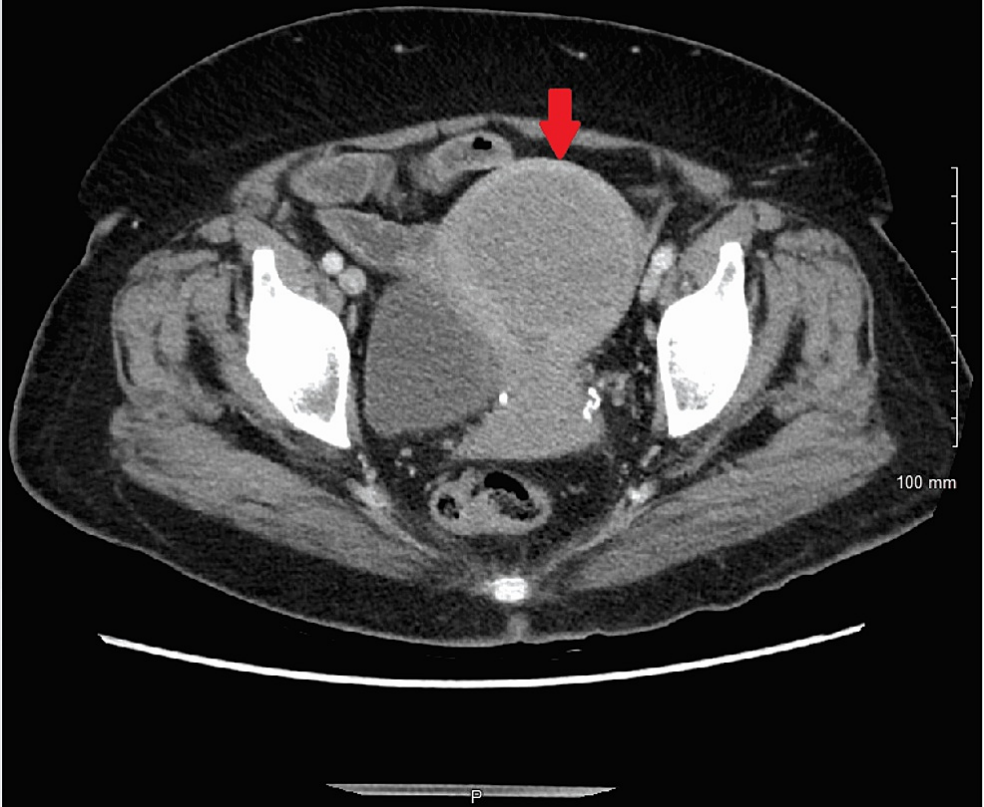
Hypodense nodule approximately 5.6 cm × 6.2 cm in size noted on computed tomography of the abdomen and pelvis, likely a uterine fibroid.

**Figure 3:**
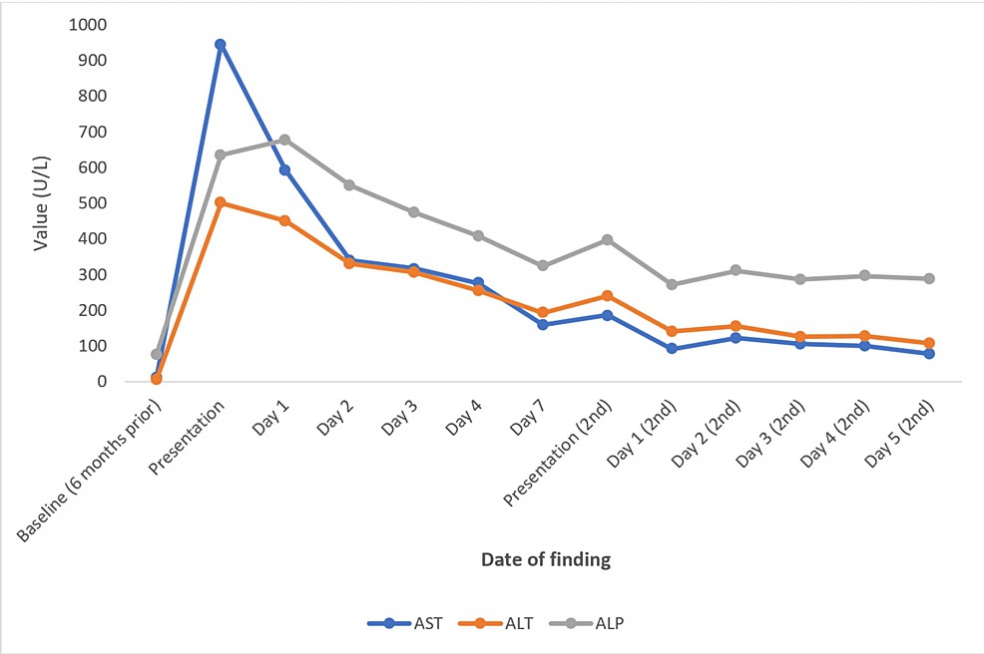
Trend of patient’s LFTs across two hospitalizations. AST: aspartate aminotransferase; ALT: alanine transaminase; ALP: alkaline phosphatase; LFTs: liver function tests

Upon arriving at the emergency room, the patient was afebrile with a temperature of 99.5°F and tachycardic to 115 beats per minute with an oxygen saturation of 95% on room air. The physical examination was significant for scleral icterus, full-body jaundice, bilateral wheezing in all lung fields, and rhonchi noted in the bilateral lower lung fields. Initial labs were significant for aspartate aminotransferase (AST) of 945 U/L, alanine transaminase (ALT) of 502 U/L, alkaline phosphatase (ALP) of 636 U/L, total bilirubin of 11.9 mg/dL, direct bilirubin of 9.9 mg/dL, total protein of 7.3 g/dL, albumin of 2.8 g/dL, prothrombin time/international normalized ratio (PT/INR) of 16.2 seconds/1.4, activated partial thromboplastin time (aPTT) of 31.4 seconds, white blood cell (WBC) count of 11,930/µL, and a D-dimer of 389 ng/mL. The patient also tested negative for coronavirus disease 2019. Chest X-ray and ultrasound of the liver showed no acute abnormalities. A computed tomography (CT) of the abdomen and pelvis with contrast showed a hypodense nodule involving the uterus measuring 5.6 cm × 6.2 cm, suspicious for a uterine fibroid otherwise no acute intra-abdominal process (Figures 1, 2). A CT angiogram of the chest was also obtained due to the patient’s mildly elevated D-dimer which showed no evidence of pulmonary embolism. The patient was given methylprednisolone 40 mg intravenously (IV) and three nebulizer treatments of ipratropium-albuterol 0.5-2.5 mg/3 mL solution with clinical approvement. The patient was then admitted to the medicine service for management of her asthma exacerbation and further workup of her transaminitis of unknown origin. During medication reconciliation, it was noted that the patient had been taking atorvastatin 20 mg daily for the management of her hyperlipidemia for 11 months prior to her admission. Atorvastatin was discontinued considering her newly diagnosed transaminitis. A detailed chart review showed that her baseline LFTs from six months ago were normal with AST of 13 U/L, ALT of 7 U/L, ALP of 77 U/L, and total bilirubin of 0.6 mg/dL (Table 1, Figure 3).

**Table 1:** Trend of patient’s hepatic function panel across two hospitalizations. AST: aspartate aminotransferase; ALT: alanine transaminase; ALP: alkaline phosphatase

Date	AST (U/L)	ALT (U/L)	ALP (U/L)	Total bilirubin (mg/dL)	Direct bilirubin (mg/dL)	Total protein (g/dL)	Albumin (g/dL)
Baseline six months prior to admission	13	7	77	0.6	N/A	6.9	4
Presentation	945	502	636	11.9	9.9	7.3	2.8
Day one	593	451	679	13	10.7	7.7	2.8
Day two	340	331	551	11.5	9.7	6.9	2.6
Day three	318	308	475	10.6	8.6	6.6	2.7
Day four	277	256	409	8.5	6.9	6	2.5
Day seven	160	194	325	6	4.7	5.5	2.3
Presentation (second)	187	241	398	8.6	5.6	7.2	3
Day one (second)	92	142	272	4.6	3.3	5.1	2.3
Day two (second)	123	156	312	4.7	3.1	6	2.8
Day three (second)	106	126	287	3.9	2.7	5.4	2.4
Day four (second)	101	129	297	3.8	2.5	5.7	2.6
Day five (second)	78	108	290	3.2	2.2	5.6	2.5

During the patient’s initial course, she was started on a budesonide-formoterol inhaler 160-4.5 µg/act every 12 hours and switched from IV methylprednisolone 40 mg to oral prednisone 40 mg daily for the management of her asthma exacerbation. Gastroenterology (GI) was consulted and recommended viral hepatitis, metabolic hepatitis, and autoimmune panels along with testing for Epstein-Barr virus (EBV), varicella-zoster virus (VZV), cytomegalovirus (CMV), and herpes simplex virus (HSV). GI also recommended a magnetic resonance cholangiopancreatography (MRCP) to be performed which was negative for significant intrahepatic or extrahepatic biliary ductal dilatation in addition to no choledocholithiasis. The hospital course was complicated by constipation, urinary retention, and acute urinary tract infection (UTI) with culture growing *Klebsiella pneumoniae*. By the end of day three, hepatitis and autoimmune serologies were finalized which showed ceruloplasmin within normal limits, negative for LKM antibodies (Abs), hepatitis A Ab IgM, hepatitis B surface Ag, hepatitis C Ab, and hepatitis E Ab, but were positive for an ANA titer of 1:320 and an ASMA titer of 1:40. The patient’s LFTs were down-trended to AST of 318 U/L, ALT of 308 U/L, ALP of 475 U/L, and total bilirubin of 10.6 mg/dL (Figure 3, Table 1). There was a suspicion by the primary team for AIH, so the patient’s oral prednisone was increased to 60 mg daily. GI later commented that the patient’s transaminitis was likely due to DILI from her recent use of azithromycin (and history of atorvastatin use) because the ASMA titer was not high enough for a confirmed diagnosis of AIH. The patient required outpatient GI follow-up within six weeks. On day seven, the patient no longer had a cough, was able to pass a trial of void, and continued to have down-trending LFTs. She was discharged on oral ciprofloxacin 500 mg every 12 hours for another 10 days to treat her UTI. The patient was also given a prednisone taper for the next six days starting with 30 mg daily.

**Figure 4:**
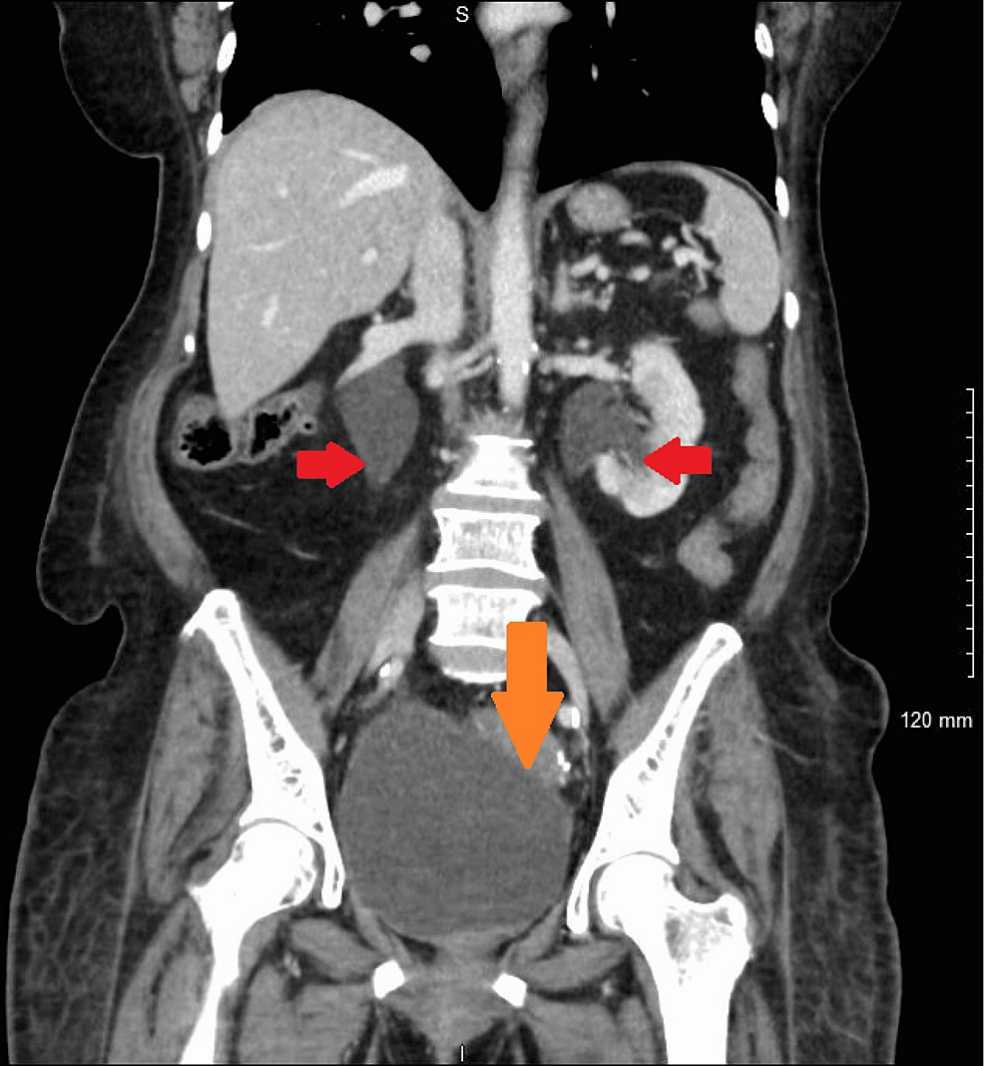
Bilateral hydroureteronephrosis (red arrows) and distended bladder (orange arrow) noted on computed tomography of the abdomen and pelvis.

**Figure 5:**
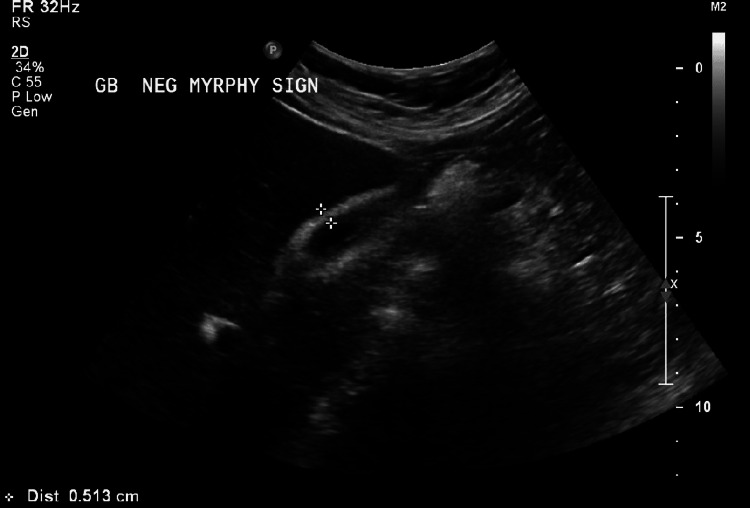
Gallbladder wall thickness of 5 mm with no gallstones noted on ultrasound.

The patient was readmitted two days later for persistent UTI and urinary retention with acute kidney injury (AKI) because she was unable to pick up antibiotics from her pharmacy. The patient’s vital signs were stable, and a physical examination was positive for bilateral costovertebral angle (CVA) tenderness, suprapubic tenderness, and suprapubic distention. CT of the abdomen and pelvis showed bilateral hydroureteronephrosis, distended bladder, and a 7 cm mass on the superior aspect of the uterus, likely a uterine fibroid (Figure 4). A foley catheter was placed and the patient was started on oral ciprofloxacin 500 mg every 12 hours for management of her UTI. The patient was also continued on her steroid taper of oral prednisone 20 mg daily followed by another two days of prednisone 10 mg daily. Urology was consulted for the patient’s acute urinary retention and AKI and recommended outpatient follow-up along with straight catheter education at home. Obstetrics and Gynecology (OBGYN) was consulted on whether the patient’s uterine fibroid may be causing her acute urinary retention but concluded that due to the location of the fibroid, it is unlikely that it is causing urinary retention and recommended outpatient follow-up at the gynecology clinic. The patient’s LFTs continued to downtrend slightly but remained above the upper limit of normal (Figure 3, Table 1). The viral labs from the previous hospitalization returned as follows: EBV DNA by polymerase chain reaction (PCR) was undetectable, VZV by anti-complement immunofluorescence (ACIF) was greater than or equal to 1:4 (evidence for immunity), HSV type 1 IgM was negative, HSV type 2 IgM was negative, and CMV DNA by PCR was undetectable. Gallbladder ultrasound showed a contracted gallbladder wall with a gallbladder wall thickness of 5 mm but no gallstones or common bile duct dilation (Figure 5). Repeat fasting abdominal ultrasound on day two of her second hospital admission showed no acute findings and resolved hydronephrosis. An ANA titer was repeated during this hospitalization which was negative. IgG levels were 1,296 mg/dL, which was within normal limits. GI recommended interventional radiology (IR)-guided liver biopsy to confirm the patient’s hepatobiliary pathology. The patient was discharged on day six after completing her liver biopsy and clinical improvement of her UTI and AKI. The patient will be taking oral cefpodoxime 200 mg twice daily for four days to complete her UTI treatment. The patient will be following up with OBGYN, urology, and GI as an outpatient. The liver biopsy results showed mild portal inflammation consisting mainly of lymphocytes with rare plasma cells and eosinophils with mild interface hepatitis, marked lobular disarray characterized by numerous necroinflammatory infiltrates including plasma cells, apoptotic bodies, hepatocytes undergoing ballooning degeneration, activation of sinusoidal lining cells with sinusoidal infiltrates, and aggregates of ceroid-containing macrophages. Bile duct and mild parenchymal cholestasis were also noted. The trichrome stain showed no significant fibrosis. These findings were consistent with acute hepatitis with moderate activity, favoring DILI, mixed hepatic and cholestatic type.

## Discussion

Although our patient had some of the signs of AIH, not enough of the diagnostic criteria, as outlined by the International Autoimmune Hepatitis Group (IAIHG), were fulfilled for a confirmed diagnosis of AIH [17]. During the initial hospitalization, a strong ANA titer of 1:320 was found along with a mildly positive ASMA titer of 1:40. However, during rehospitalization, the ANA titer became negative. ANA titers have been shown to decrease after treatment of systemic autoimmune rheumatic diseases [18]. ANA has also been shown to be positive in healthy patients, so it is a nonspecific diagnostic marker [19]. A liver biopsy later confirmed a diagnosis of acute hepatitis due to DILI. Although rare plasma cells and mild interface hepatitis were noted, due to the presence of cholestasis and bile duct damage in addition to the lack of significant fibrosis, the pathology results favored DILI rather than AIH [6,15]. Azithromycin was implicated as the most likely culprit in this case of DILI because it was the only new medication that the patient took recently with this documented side effect [6].

Azithromycin-induced liver injury usually occurs within one to three weeks after drug initiation and is predominately hepatocellular or cholestatic in nature [6]. Cholestatic DILI typically takes longer to resolve when compared to hepatocellular DILI; however, patients with hepatocellular DILI do not fare as well with 9% of patients resulting in fatality or liver transplant [11]. Azithromycin has been reported to cause ALT elevations in 1-2% of the population; however, the mechanism behind azithromycin-induced liver injury is poorly understood [20]. Our patient had signs of azithromycin-induced liver injury one month after drug initiation and was more cholestatic in nature, with an ALT/ALP ratio of <2. DILI may resemble AIH and respond to corticosteroids, which was the case for our patient [11]. Minocycline, nitrofurantoin, and infliximab have been the agents most commonly incriminated with this drug-induced AIH-like injury [15]. However, there are no randomized controlled trials supporting the use of steroid therapy for the treatment of DILI [11]. The mainstay treatment of azithromycin-induced liver injury remains withdrawal of the medication; however, there is a small possibility that liver transplantation may be required [6].

In previous studies, azithromycin was noted to cause DILI in a higher proportion of patients who had pre-existing liver disease compared to those without a history of liver disease [10]. Our patient had no personal or family history of liver disease prior to her admission which makes her presentation rarer. She was taking atorvastatin, which has been shown to have hepatotoxic effects; however, she was taking that medication 11 months prior to her admission, and baseline LFTs six months prior were within normal limits [21]. Azithromycin is a relatively safe antibiotic that has a wide range of uses but it is important to note that DILI is one of its rare and potentially fatal side effects [1,6].

## Conclusions

DILI and AIH may have similar clinical presentations. Detailed history taking and extensive laboratory testing are necessary to differentiate between these conditions. Our patient was initially suspected to have AIH but was later confirmed with a liver biopsy to have acute hepatitis secondary to DILI with a temporal relationship to azithromycin. Azithromycin remains a cornerstone treatment for many bacterial infections in current clinical practice, but it is important to highlight one of its rare and potentially fatal side effects, azithromycin-induced liver injury.
